# A Mathematical Model of Muscle Containing Heterogeneous Half-Sarcomeres Exhibits Residual Force Enhancement

**DOI:** 10.1371/journal.pcbi.1002156

**Published:** 2011-09-29

**Authors:** Stuart G. Campbell, P. Chris Hatfield, Kenneth S. Campbell

**Affiliations:** Department of Physiology and the Center for Muscle Biology, University of Kentucky, Lexington, Kentucky, United States of America; University of Calgary, Canada

## Abstract

A skeletal muscle fiber that is stimulated to contract and then stretched from L_1_ to L_2_ produces more force after the initial transient decays than if it is stimulated at L_2_. This behavior has been well studied experimentally, and is known as residual force enhancement. The underlying mechanism remains controversial. We hypothesized that residual force enhancement could reflect mechanical interactions between heterogeneous half-sarcomeres. To test this hypothesis, we subjected a computational model of interacting heterogeneous half-sarcomeres to the same activation and stretch protocols that produce residual force enhancement in real preparations. Following a transient period of elevated force associated with active stretching, the model predicted a slowly decaying force enhancement lasting >30 seconds after stretch. Enhancement was on the order of 13% above isometric tension at the post-stretch muscle length, which agrees well with experimental measurements. Force enhancement in the model was proportional to stretch magnitude but did not depend strongly on the velocity of stretch, also in agreement with experiments. Even small variability in the strength of half-sarcomeres (2.1% standard deviation, normally distributed) was sufficient to produce a 5% force enhancement over isometric tension. Analysis of the model suggests that heterogeneity in half-sarcomeres leads to residual force enhancement by storing strain energy introduced during active stretch in distributions of bound cross-bridges. Complex interactions between the heterogeneous half-sarcomeres then dissipate this stored energy at a rate much slower than isolated cross-bridges would cycle. Given the variations in half-sarcomere length that have been observed in real muscle preparations and the stochastic variability inherent in all biological systems, half-sarcomere heterogeneity cannot be excluded as a contributing source of residual force enhancement.

## Author Summary

Textbooks often state that the force produced by a contracting muscle depends on its length. Nearly 60 years ago, it was discovered that this length-tension relationship is violated if the muscle is stretched to a given length while contracting, in which case the muscle produces more force than if it was stretched to the given length prior to contraction. This effect is known as residual force enhancement, and its mechanism remains controversial. Understanding residual force enhancement is important because it potentially affects outcomes of many *in vivo* and *in vitro* experiments where contracting muscles or muscle preparations are stretched. In this work, we use a computational model of half-sarcomeres connected in series to show that residual force enhancement is an emergent behavior of the contractile system when the half-sarcomeres are not completely identical. Force enhancement in the model shares several key properties with the phenomenon observed in real muscle, including independence from the rate of stretch and proportionality to stretch magnitude. Enhancement in the model is produced by a previously undescribed mechanism in which complex interactions between the heterogeneous half-sarcomeres dissipate strain-energy from the imposed stretch at very slow rates, creating a long-lasting, enhanced level of force.

## Introduction

Stretching a contracting muscle produces a dynamic, non-linear force response. Some features of the response can be explained by classical cross-bridge theory [1], [2], but others apparently cannot. Residual force enhancement following stretch is a phenomenon widely believed to fall into the latter category [3], [4].

**Figure 1:**
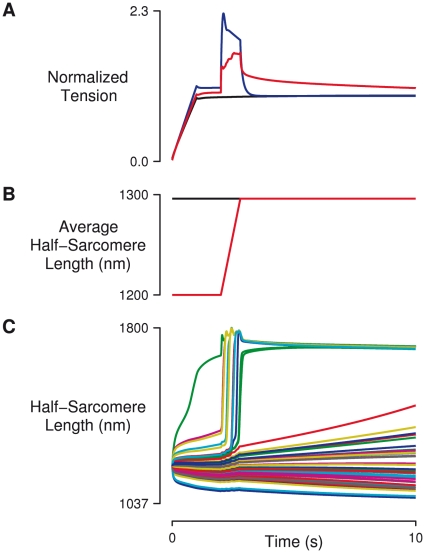
Basic stretch responses. (A) Isometric (black trace) and stretched (red trace) tension responses in a myofibril consisting of 50 half-sarcomeres in series, with 20% variation in half-sarcomere strength (*α* = 0.2). The stretch response in a single half-sarcomere (which is equivalent to a multi half-sarcomere system with no variation in half-sarcomere strength) is also shown for comparison (blue trace). (B) Time course of average half-sarcomere length for isometric (black trace) and stretch (red trace) protocols. In each simulation, fibers were activated gradually over a period of one second, and then allowed to stabilize for another second before the stretch was imposed. Tension in the myofibril consisting of a single half-sarcomere decays rapidly back to the isometric value, while tension in the heterogeneous fiber remains elevated for many seconds following stretch, strongly resembling the classical force enhancement response. (C) Time courses of 50 individual half-sarcomeres are shown for a single simulation with an *α* value of 0.2, and reveal continuing internal motion after the end of stretch. This is the source of enhancement.

The basic experimental finding was first described by Abbott & Aubert [5]. They found that a dogfish muscle stretched during active contraction produced greater steady-state force than if stretched to the same length prior to activation. (A stretch protocol of this kind is depicted in Figure 1.) This was seen as a departure from the well known steady-state length tension relationship as the muscle could be made to produce different amounts of force at a given length by changing its history of movement. Since Abbott & Aubert's initial description, force enhancement has been observed in many other species, muscle types, and experimental preparations (reviewed elsewhere [6]).

Researchers have been studying residual force enhancement for nearly sixty years, but the underlying mechanism remains unknown and controversial. No existing models of muscle fully explain the observed behavior, and development of a model that does could therefore improve biomechanical analyses of musculoskeletal systems. Such a model would also be helpful to the growing number of investigators who seek to use mechanical measurements on muscle tissue to elucidate the integrative function of individual sarcomeric proteins [7], [8], [9]. If the effects of force enhancement are present in the data sets they collect but not in the mathematical models that they use to analyze them, the results are unlikely to be interpreted correctly.

Several hypotheses have been advanced to explain residual force enhancement. The potential role of titin has been highlighted by Bagni and co-workers [10], [11], [12], but classically the sarcomere length heterogeneity mechanism has received the greatest amount of attention [6], [13], [14]. This was developed by Morgan and co-workers following experimental studies and the development of mathematical theories [13], [15]. Their hypothesis considers a muscle with an average sarcomere length on the descending limb of the steady-state length-tension curve, and assumes that there is some amount of heterogeneity in the initial length of each sarcomere. As the muscle is stretched, longer (weaker) sarcomeres lengthen more rapidly than shorter (stronger) ones, reach a yield point, and then lengthen uncontrollably until tension is determined almost entirely by passive elements. This sudden lengthening or ‘popping’ of weak sarcomeres allows serially connected sarcomeres to stretch less than they otherwise would. They thus produce more force because they remain at a sarcomere length that is shorter than the average sarcomere in the muscle, and are therefore positioned close to the isometric plateau. The net effect is that tension developed by the complete muscle system remains elevated following active stretch.

While several studies have offered evidence of sarcomere heterogeneity in muscle preparations [14], [16], the claim that it is the principal source of residual force following stretch remains controversial. Two experimental results have been used against the sarcomere popping hypothesis: first, that enhancement is seen at muscle lengths on the ascending and plateau portions of the length-tension curve, and second, that enhanced force after stretch can exceed the isometric force at optimal length [3]. Neither of these results are possible under the sarcomere popping hypothesis as it stands [13]. Furthermore, the notion that residual force enhancement requires multiple sarcomeres has been directly challenged by new experiments showing enhancement in a single-sarcomere myofibrillar preparation [17].

To date, most computational models of interacting heterogeneous sarcomeres have been based on the classic isometric length-tension curve and Hill-type force-velocity relations [13], [18]. In this work, we investigate residual force enhancement using a biophysically-detailed computational model of heterogeneous half-sarcomeres that was originally developed for a different purpose [19]. Rather than empirical length-tension and force-velocity relations, this model calculates force produced by each half-sarcomere from equations representing a population of actin myosin cross-bridges cycling through a strain-dependent kinetic scheme. The simulations show enhancement after stretch as a consequence of half-sarcomere inhomogeneity, but the effect is produced by a mechanism that is distinct from that of (half-) sarcomeres ‘popping’ to the passive length-tension curve.

## Methods

### Model of interacting half-sarcomeres

A model of mechanically-coupled half-sarcomeres was previously described by one of us [19]. Full details of the model, including equations and parameter listings can be found in that publication and its accompanying supplementary information. Unless otherwise noted, the parameters used in the current simulations were the same as those obtained in previous work [19] by fitting simulated records to experimental data recorded using chemically permeabilized rabbit psoas fibers at 15°C [20].

The model is based on equations that define the length- and time-dependent force produced by individual half-sarcomeres. The total number of myosin cross-bridges available to participate in force production (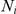) within a single half-sarcomere *i* having length 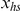 is given by the equation
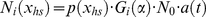
(1)

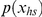
, the proportion of myosin heads at a given half-sarcomere length that are available to produce force, is based on the length-dependent overlap of thick and thin filaments. The average number of myosin heads per unit cross-sectional area in a half-sarcomere (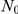) was 1.15×10^17^ m^−2^ and is scaled by 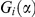, a variable that is randomly selected from a Gaussian distribution with a variance of *α* and a mean value of unity. Unless otherwise noted, *α* was set to 0.2 for all simulations. Because this quantity cannot be directly measured in experiments, sensitivity of model results to the value of *α* was assessed in a series of simulations (see below). The only change in Equation 1 from the previously published model is the addition of the term 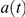, which represents the fraction of actin binding sites activated by Ca^2+^ at time *t*. This term, which is identical at any given time for all half-sarcomeres in the model, improves the numerical stability of calculations by allowing non-instantaneous activations.

The force produced by cross-bridges in each half-sarcomere is determined by analyzing cross-bridge population distributions as originally described by Huxley [1]. The number of attached cross-bridges is defined by a kinetic model consisting of detached (D), attached pre-powerstroke (A_1_), and attached post-powerstroke (A_2_) states. Transitions between states have strain-dependent kinetic rates [20]. Passive force generated by a single half-sarcomere is assumed to follow an exponential dependence on half-sarcomere length as follows:
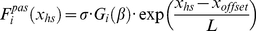
(2)The parameters 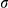, 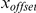, and *L* were determined by fitting passive length-tension measurements from rabbit psoas preparations [21], [22]. Values are given in Table 1. The term 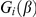 is the second addition to the previously published model, and represents a normally distributed random number having a mean value of unity and variance *β*. It can be used to introduce variability into the passive stiffness of half-sarcomeres in the same manner as *α* introduces heterogeneity in active properties. *β* was set to zero for all simulations, except where noted. The total force produced by a half-sarcomere is the sum of passive and active (cross-bridge based) forces.

**Table 1:** Passive length-tension parameter values, obtained by fitting Equation 2 to published data from rabbit psoas muscle [21], [22].

Parameter	Value	Units
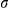	112	N m^−2^
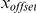	625	nm
*L*	136	nm

Most simulations use 50 half-sarcomeres connected in series, with exceptions noted in the text. The length of the complete fiber is set by the user for each time-point in the simulation while the lengths of the individual half-sarcomeres are calculated by the computer by balancing the force at each connection between half sarcomeres.

### Residual force enhancement simulation protocol

The simulation procedures mimicked published experimental protocols [23]. Trials were run in pairs. At the beginning of the first simulation, 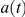 was linearly increased from 0 to 1 over the first second to gradually activate the half-sarcomeres. The overall length of the network of coupled half-sarcomeres was held isometric at an initial length *L*_0_ during the period of increasing activation and for 1 second afterward, at which point the system was stretched at constant velocity to the final length, *L*_final_. In the companion simulation, the muscle was activated at *L*_final_ and held at that length thereafter.

The magnitude of the force enhancement was defined as the percentage increase above the isometric force, i.e.
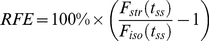
(3)where 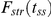 and 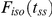 are the forces produced in the ‘ramp and hold’ and isometric simulations, respectively, at time 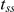 after the end of stretch. Since real muscle cannot be activated indefinitely without fatiguing (in the case of electrically-excitable fibers) or ‘running-down’ (in the case of chemically permeabilized preparations), 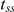 was set equal to 6 seconds after the conclusion of length change for all values of RFE reported herein. This is at least as large as the 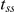 values used in many experiments (e.g. a *t*_ss_ value of 4 seconds is used in ref. [24]).

Unless noted otherwise, the length change protocol was an 8% stretch performed at 0.1 *L*_0_ s^-1^, starting from an average half-sarcomere length of 1200 nm. Tension traces shown in each figure are the average of 60 simulations run with different random number seeds to produce distinct patterns of half-sarcomere variation. Averaging reduced the stochastic noise introduced by random assignment of the number of myosin heads per unit cross-sectional area in each half-sarcomere.

## Results

### Half-sarcomere heterogeneity causes a slow component of force decay following stretch

A typical simulated force response to a ramp-and-hold length change for a single half sarcomere is shown by the blue trace in Figure 1A. There is a large tension transient at the start of stretch, after which tension falls even though the fiber is still lengthening. When the stretch ceases, tension decays almost immediately to the isometric (steady-state) value. When 50 dissimilar half-sarcomeres are arranged in series and stretched, the tension response is altered greatly (red trace, Figure 1A). After an initial rapid rise, tension continues to increase throughout the stretch. When the stretch stops, tension begins to drop but remains elevated above the corresponding isometric tension. Variation in the number of cycling cross-bridges per half-sarcomere caused half-sarcomere lengths to diverge substantially before and after stretch, with some lengthening rapidly during the applied length change (Figure 1C, also see Video S1). The slowly dissipating phase of the tension response seen in the heterogeneous case strongly resembles the residual force enhancement observed experimentally.

### RFE is independent of stretch velocity and linearly dependent on stretch magnitude

**Figure 2:**
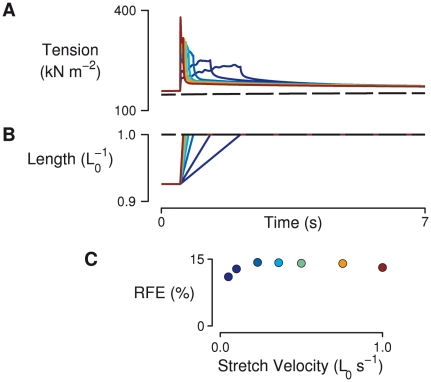
Velocity dependence of the force enhancement response. (A) Tension responses in a simulated fiber containing 50 half-sarcomeres in series and an *α* value of 0.2 at various stretch velocities. The dashed trace represents tension under isometric conditions at the final length (*L*_0_). (B) Fiber length as a fraction of final length (*L*_0_). (C) Residual force enhancement magnitude (RFE) at each of the simulated stretch velocities. RFE was not strongly dependent on stretch velocity. Colors in each panel link tension, length, and RFE values that have the same stretch velocity.

When stretches of different velocities but equal magnitude were imposed on the heterogeneous model (Figure 2), residual force enhancement was nearly constant over the entire range of stretch velocities, in agreement with published reports [23], [25].

**Figure 3:**
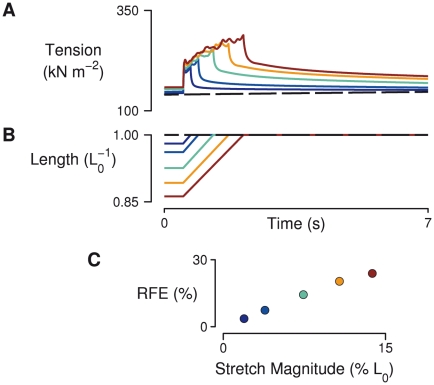
Stretch magnitude dependence of the force enhancement response. (A) Tension responses in a simulated fiber containing 50 half-sarcomeres in series and an *α* value of 0.2 for various stretch magnitudes. The dashed trace represents tension under isometric conditions at the final length (*L*_0_). (B) Fiber length as a fraction of final length (*L*_0_). (C) Residual force enhancement magnitude (RFE) at each of the simulated stretch magnitudes. RFE was proportional to stretch magnitude, in agreement with many experimental measurements. Colors in each panel link tension, length, and RFE values that have the same stretch magnitude.

The dependence of stretch magnitude was also examined, using a range of stretch sizes (2–16% *L*_0_), each with a stretch velocity of 0.1%
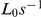
(Figure 3). RFE was proportional to the magnitude of stretch, a feature that has also been reported for real muscle preparations [5], [25], [26].

### RFE is present at varying degrees of myofilament overlap

**Figure 4:**
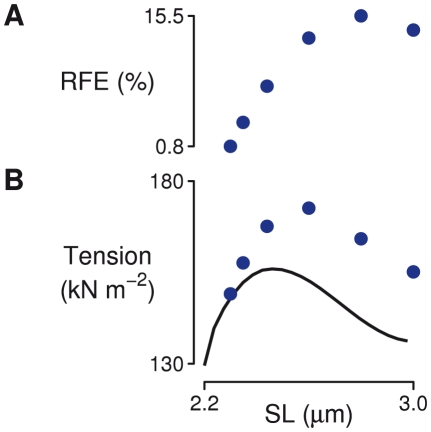
Force enhancement at different degrees of myofilament overlap. (A) Residual force enhancement (RFE) as a function of final mean sarcomere length, simulated in a fiber containing 50 half-sarcomeres in series and an *α* value of 0.2. (B) Enhanced tension after stretch (circles) shown with the steady-state length tension curve produced by the model (solid line). Some enhancement can be seen on the ascending limb, plateau, and descending limb of the length tension curve.

Isometric simulations at many half-sarcomere lengths were performed to construct a steady-state length tension curve for default model parameters. This defined half-sarcomere length intervals for the ascending limb, plateau, and descending limb of the length-tension relation (Figure 4b). The stretch protocol was then performed at average half-sarcomere lengths of 1134 and 1160 nm (ascending limb), 1210 and 1296 nm (approximate plateau), and 1404 and 1512 nm (descending limb). A small amount of enhancement was seen on the ascending limb of the length-tension relation (0.8–3.5%, Figure 4a), compared with 2–5% observed experimentally on the ascending limb in frog muscle [27]. 8–13% enhancement is produced from stretches ending at lengths on the plateau of the length-tension curve, demonstrating that the model is capable of producing residual force enhancement that exceeds the force at optimal length (Figure 4b). Among the initial lengths tested, RFE was greatest for stretches on the descending limb.

### Small amounts of heterogeneity give rise to enhancement

**Figure 5:**
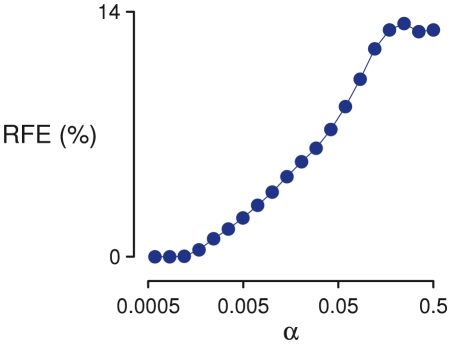
Sensitivity of residual force enhancement to amount of half-sarcomere heterogeneity. Residual force enhancement in a fiber composed of 50 half-sarcomeres in series as a function of *α*, the variation in half-sarcomere strength. Stretch magnitude was 8% of the final length. Note the logarithmic scale on the x-axis. As little as 2% variation in half-sarcomere strength produces 5% residual force enhancement following stretch.

The above results were obtained using *α* = 0.2 (±20% variation in the active tension capacity of half-sarcomeres). However, this level of heterogeneity is not required for the appearance of enhancement behavior. Repeating the stretch protocol over a wide range of *α* values shows that RFE is highly sensitive to even small amounts of variation (Figure 5), with an *α* value of 0.02 producing more than 5% RFE. Enhancement essentially saturates at *α* values exceeding 0.1 (Figure 5).

### Heterogeneous passive properties also produce enhancement

**Figure 6:**
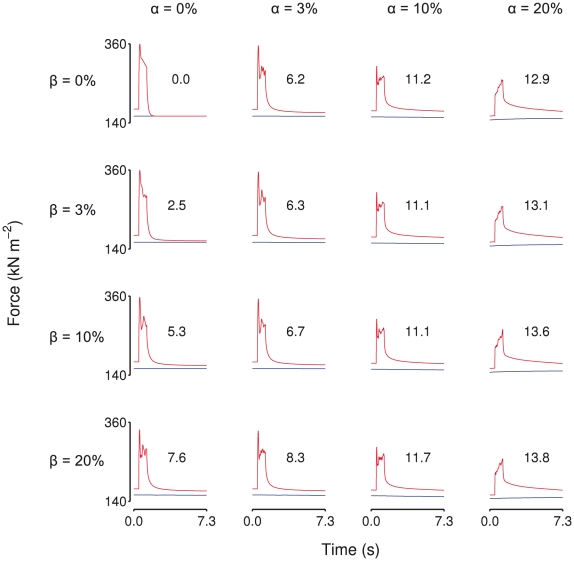
Heterogeneous passive tension properties also produce enhancement. The standard stretch protocol was simulated with varying levels of passive (*β*) and active (*α*) variation among 50 half-sarcomeres connected in series. Each plot shows tension responses in stretch (red) and isometric conditions (blue). Inset numbers indicate the percentage of residual force enhancement after stretch for the given combination of *β* and *α* shown in the margins. Increasing just passive variation among half-sarcomeres (first column) gives rise to residual force enhancement behavior. When *α* and *β* are co-varied, it is apparent that the magnitude of enhancement is more sensitive to *α*.

Simulations were also used to test whether variability in the passive elastic properties of half-sarcomeres (quantified by the parameter *β*) produced residual force enhancement. RFE was roughly half as sensitive to *β* as to *α* (Figure 6). Under the conditions of the standard stretch protocol, the maximal enhancement produced by passive tension variation was 7.6%. *β* in combination with *α* did not increase RFE substantially. With *α* at 0.2, the addition of *β* = 0.2 raised RFE only slightly, from 12.9% to 13.8%.

### Enhancement occurs even in conditions that stabilize sarcomeres

**Figure 7:**
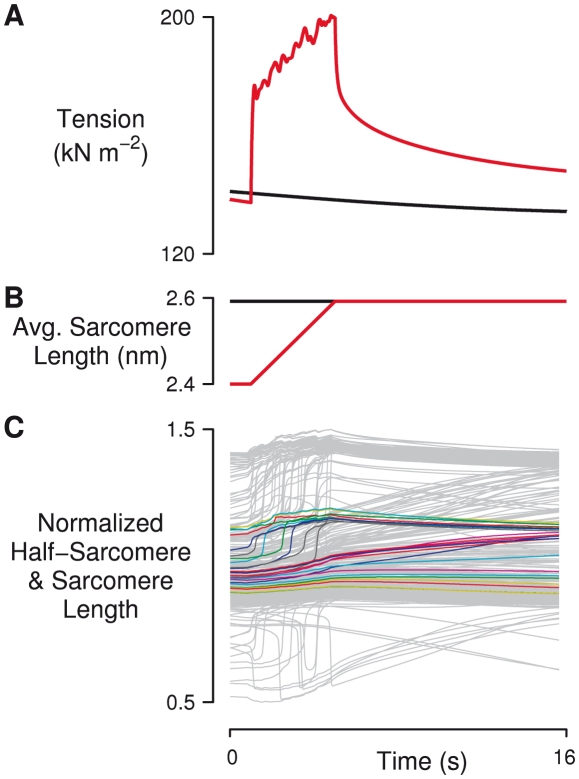
Force enhancement in a model with multiple myofibrils. A model consisting of 6 myofibrils in parallel, each containing 50 half-sarcomeres in series was subjected to a slow ramp stretch (8% *L*_0_ over 4 seconds). Z-lines in adjacent myofibrils were mechanically linked so that they stayed in register throughout the protocol, while half-sarcomeres remained unrestricted. *α* was set to 0.2. (A) Tension in the stretched muscle (red) decayed slowly and remained above the isometric control record (black), giving 12.7% residual force enhancement 6 seconds post-stretch. (B) Average sarcomere length during the two simulations (colors as in panel A). (C) Sarcomere length for each of 25 whole sarcomeres in the simulated muscle fiber (color traces), normalized to the initial average sarcomere length of 2.4 µm. The relative stability and absence of ‘popping’ in sarcomeres is seen by comparison with the time course of half-sarcomere lengths (gray traces, normalized to initial half-sarcomere length of 1.2 µm) appearing in the background.

In intact muscle, structural proteins such as desmin are thought to link z-disks of adjacent myofibrils [28]. While this wouldn't be expected to affect half-sarcomere length variability, it could stabilize the lengths of whole sarcomeres across (and thus potentially through a ‘second-order effect’ along) fibers. In a series of additional simulations, we tested the possibility that stabilization among coupled myofibrils could eliminate residual force enhancement. The model size was increased to 6 myofibrils in parallel, each with 50 half-sarcomeres in series. *k*_im_ was increased from 0 to 0.1, effectively forcing z-lines to be registered across parallel myofibrils. Using an *α* value of 0.2, the model was stretched (8% *L*_0_ over 4 seconds) and the resulting force response showed an RFE of 12.7% (Figure 7). A wide range of half-sarcomere lengths can be seen throughout the record (Figure 7C), just as in single myofibril simulations (Figure 1C). At the same time, the relative variation in sarcomere length was much smaller than the variation in half-sarcomere length, and while a few of the whole sarcomeres elongate rapidly during stretch, they do not ‘pop’ to a length dominated by passive tension as some half-sarcomeres do (Figure 7C).

## Discussion

In this work, we used a computer model of muscle fibers previously developed for another purpose to show that enhanced force after stretch is an emergent property of dissimilar half-sarcomeres. The relevance of this result to real muscle physiology is supported by good agreement between modeled and measured phenomena. Two widely reported properties of force enhancement are its independence from stretch velocity and its linear dependence on the magnitude of stretch (see review by Herzog *et al.* [3]). Residual force enhancement exhibited by the model shares these same properties (Figures 2 and 3). In the model, RFE was related to percent stretch magnitude by a factor of 1.75, compared with a value of 1.83 observed in intact frog skeletal muscle fibers (calculated from data in ref. [29]). Hence, the amount of enhancement seen in the model is comparable to typical values reported for intact muscle.

In a comprehensive review, Herzog *et al.* [3] note two primary limitations to the theory that half-sarcomere heterogeneity (and more specifically sarcomere popping) underlies residual force enhancement. The first is that this explanation only holds for half-sarcomere lengths on the descending limb of the length-tension relationship, while small but consistent amounts of residual force enhancement can be seen at lengths corresponding to the ascending and plateau regions of the relation [27]. The second limitation is that it does not predict enhanced forces greater than the force produced at optimal isometric length, whereas supra-optimal tensions following stretch have been reported in experimental preparations [16], [24]. The model used here predicts enhancement after stretch on each portion of the length-tension curve (Figure 4), which demonstrates that half-sarcomere heterogeneity as represented here does not suffer from the same limitations assigned to earlier theory.

While non-uniformity is the basis for enhancement in the present model, the mechanism at work is not one of ‘popping’ sarcomeres as proposed and modeled by Morgan [13]. According to that theory, force enhancement after stretch occurs when weak sarcomeres ‘pop’ or elongate to a point where they cannot produce active tension and passive structures cause force to increase with stretch. As these weak sarcomeres absorb most of the applied length change they leave stronger sarcomeres to stretch less, loose less myofilament overlap, and remain at higher active tensions. The net effect is a permanent enhancement of force. The mechanism of Morgan *et al.* reflects the muscle's steady-state length tension relationship, but the emergent behavior described in this work results from more complex, dynamic processes.

**Figure 8:**
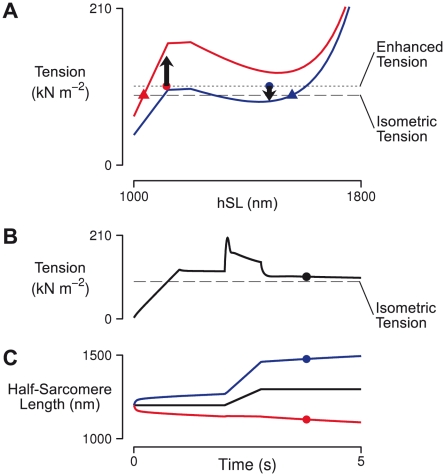
Enhancement by dynamic interactions among dissimilar half-sarcomeres. Residual force enhancement is produced here in a model of two dissimilar half-sarcomeres connected in series. (A) The length-tension coordinates of the two half-sarcomeres are shown here one second after the end of stretch (circles) and at steady-state (triangles, >30 seconds post stretch). The steady-state length-tension curves, which include passive and active tension, are shown for both half-sarcomeres, along with horizontal lines indicating levels of isometric and enhanced tension for the system. (B) Tension response to stretch (solid trace). The circle marks the time point corresponding to the length-tension coordinates marked by circles in panel A. (C) Time courses of average (black trace) and individual half-sarcomere lengths (red and blue traces). Circles correspond with those plotted in panel A.

Figure 8 and Video S2 show how residual force enhancement is produced in our model. This simulation consists of two dissimilar half-sarcomeres connected in series, one having fewer crossbridges (the ‘weaker’ half-sarcomere) than the other (the ‘stronger’ half-sarcomere). When this system is stretched, the weaker unit absorbs the applied stretch, skewing its distributions of attached cross-bridges. This positive shift in cross-bridge strain elevates tension in the weaker half-sarcomere *above* the level predicted by its steady-state length tension curve (Figure 8A). As the weaker half-sarcomere lengthens, the strong one shortens, lowering the mean strain in its cross-bridge population and causing tension to fall *below* steady-state. The populations of cross-bridges cycling within each modeled half-sarcomere seek to return to their steady-state conditions as non-equilibrium cross-bridges detach and re-attach at strain values characteristic for an isometric half-sarcomere. In the case of these coupled half-sarcomeres, however, the steady-state equilibria lie in different directions. These opposing effects in the weaker and stronger half-sarcomeres cause overall muscle tension to remain steady at an elevated level because equal force across all half-sarcomeres can only be achieved by weaker and stronger half-sarcomeres lengthening and shortening respectively. This perpetuates elevated strain in the cross-bridges of the weaker half-sarcomere and reduced strain in the stronger half-sarcomere, and the effect becomes long-lasting.

Residual force enhancement produced by the model is ‘permanent’ on the practical time-scale used for experiments on intact muscle, which is limited by the facts that tetanic contractions cannot be maintained indefinitely in intact fibers and skinned preparations ‘run down’ if they are activated for long periods. However, it is interesting to note that if more than ∼30 seconds following stretch are simulated, tensions in the stretched and reference isometric cases eventually converge and become indistinguishable. Hence, the mechanism just described does not support a permanent increase in tension after stretch in the mathematical sense, unlike the popping sarcomere hypothesis [13].

It is interesting to note that similar simulation results were obtained by Telley *et al.* [30] in a model where crossbridge cycling was not explicitly represented. Instead, Telley *et al.* modeled each half-sarcomere as a contractile element (governed by classical steady-state force-velocity and force-length relations) in parallel with a viscoelastic element representing titin. They too observed residual force enhancement following stretch when the properties of half-sarcomeres were non-uniform, and movement of half-sarcomeres in their model strongly resembles that shown in our model (compare our Figure 1c with Figure 7A in ref. [30]). One might well wonder how the model of Telley *et al.* produces enhancement without a representation of crossbridge cycling if, as we claim, crossbridge dynamics are mediating the response (Figure 8). We believe that the answer lies in Telley *et al.*'s representation of titin as a viscoelastic element.

When subjected to length changes, a viscoelastic element can impart history-dependent force behavior that mimics to a first approximation the behavior exhibited by populations of cycling crossbridges under the same circumstances. This suggests that history-dependent mechanical behavior, regardless of molecular source or sources, can contribute to force enhancement following stretch. Although we have not yet modeled this situation ourselves, we suspect that Telley *et al.*'s viscoelastic approach can only produce appreciable force enhancement when the magnitude of the viscous component attributed to titin is much larger than that which is apparent during experiments performed with passive muscle fibers. A new muscle model that includes both crossbridge cycling and realistic viscoelastic passive properties would help elucidate the relative importance of each in the residual force enhancement response.

Dynamic interactions among dissimilar half-sarcomeres are manifest in our simulations by half-sarcomere movement that continues even after the end of the stretch (e.g. Figure 1C). Measurements of half-sarcomere length in single rabbit psoas myofibrils undergoing stretch show non-uniform lengths and rates of length change that are reminiscent of the dynamic interactions exhibited by our model [14], [31]. These observations serve as evidence for the presence of variability in half-sarcomere mechanical properties. Telley *et al.* [14] observed half-sarcomeres that both lengthen and shorten in the moments following stretch, which is the basis of the dynamic enhancement mechanism we describe. They found, however, that half-sarcomere length prior to stretch was a poor predictor of length and motion after stretch. That is, shorter or presumably stronger half-sarcomeres were not guaranteed to stretch less than longer ones (also noted by Joumaa *et al.* [16]). These results can be reconciled with our findings if some amount of variability in both active and passive mechanical properties of half-sarcomeres is assumed. Shorter initial half-sarcomere length could be due to higher passive tension, which would mask a lower active tension capacity. Lower active tension would be exposed during stretch when attached bridges provide most of the resistance to lengthening, and this initially ‘strong’ half-sarcomere could therefore be seen to lengthen more than apparently weaker ones.

These lines of numerical and experimental evidence suggest that tension responses to length change in contracting muscle are in large part the product of dynamic interactions between heterogeneous half-sarcomeres. Functional measurements of heterogeneous half-sarcomere length in myofibrils [14], [31] are indicative of intrinsic variations that could take many forms. Individual myosin molecules seem to be replaced in real muscles roughly once a week, which suggests that repair and replacement mechanisms could lead to half-sarcomere variability. Furthermore, actin filament lengths are variable [32], which could cause differences in the number of myosin binding sites in each half-sarcomere. Many myofilament proteins have alternate isoforms and splice variants that modulate both active (e.g. myosin, myosin light chain, troponin, etc.) and passive (e.g. titin) properties of muscle. The possibility that isoforms of one or more of these proteins could be heterogeneously expressed among half-sarcomeres is supported by observations of spatial variation of myosin isoform expression within single extraocular muscle fibers [33]. Intracellular gradients in phosphorylation of myofilament proteins could produce similar effects. We believe that all of these sources of variability, and many other such effects have the potential to contribute to force enhancement. Indeed, the simulated stretch response agrees best with experimental data when multiple types of variation are included simultaneously (e.g. Figure 6, bottom right plot). New experiments that combine measurements of half-sarcomere dynamics and studies of myofilament protein expression and posttranslational modifications may therefore be required to produce new insights into residual force enhancement.

Recent experiments by Herzog and co-workers [17] have examined force enhancement in single sarcomeres in an attempt to eliminate any potential contribution of sarcomere heterogeneity to the response. They found that a large residual force enhancement was evident even in this simplified system, which the authors cite as evidence that interactions among many half-sarcomeres are not required for force enhancement. Interpreting these results should be done with care, however, as the exact number of half-sarcomeres involved in these measurements is difficult to define. Since the cantilevers attached to the myofibril were 2.5 µm wide, they could not have attached specifically to z-lines but must have overlapped with half-sarcomeres on either side. Given the complexity of this mechanical situation, it is not clear to us whether the force recorded in such experiments only reflects the central sarcomere or whether it is influenced by the distributed nature of the attachments. It is interesting to note that even in an ideal, mechanically isolated sarcomere, simulations predict the occurrence of some force enhancement via half-sarcomere dynamics (Figure 8). Still, the model predicted enhancement (∼2%) when we simulate Herzog *et al.*'s protocol is far lower than the 185% enhancement that they report. This indicates that the dynamic interaction of dissimilar half-sarcomeres is unlikely to be the only mechanism contributing to the residual force enhancement observed in Herzog *et al.*'s measurements.

It would be interesting to test our hypothesis that force enhancement is an emergent property of dissimilar half-sarcomeres by performing an experiment with a completely uniform fiber or myofibril. Unfortunately, we do not think that this will be possible because there are many potential sources of variation, including, ultimately, stochastic variation due to random Brownian motion. An alternate test may therefore be to increase variability (rather than eliminate it) and determine the experimental effect. This could, in principle, be done using a small jet of solution containing a crossbridge inhibitor such as blebbistatin to depress active tension generation in a contiguous patch of half-sarcomeres along a skeletal muscle fiber (see ref. [34] for an example in cardiac muscle). The force enhancement protocol would be administered under these conditions and in the absence of the inhibitor. If inhibition of a small number of half-sarcomeres increased the magnitude of enhancement, it would support the hypothesis advanced in this manuscript that force enhancement is mediated by non-uniformity of connected half-sarcomeres.

### Conclusions

We have used numerical simulations to discover a novel mechanism for residual force enhancement after stretch. Enhancement was an emergent behavior of a model system that assumed variation in the mechanical properties of series-connected half-sarcomeres. Force enhancement was not produced by sarcomere popping but by dynamic interaction between dissimilar half-sarcomeres which prevented rapid dissipation of strain energy stored in populations of cycling cross-bridges. The simulations suggest that observable levels of enhancement will occur if variation in half-sarcomere properties exceeds ±2%, a condition that is hard to discount in real biological systems. Hence, without excluding other contributing mechanisms, this work suggests that dynamic interactions among dissimilar half-sarcomeres are an important component of residual force enhancement.

## Supporting Information

Video S1 **Movement of half-sarcomeres following active stretch.** This video is an animation of length-tension relations as a model of 50 half-sarcomeres in series with an *α* value of 0.2 is actively stretched. (A) The steady-state length-tension relation for each of the half-sarcomeres (colored lines) is plotted with the level of tension achieved at steady-state for an isometric contraction (dashed line). Colored dots represent length vs. tension in each of the 50 half-sarcomeres at every time point in the simulation, showing how the instantaneous length-tension deviates from steady-state values. (B) The time course of tension throughout the simulation (solid trace) with the steady-state isometric tension (dashed line) for reference. The difference between the two traces after stretch shows residual force enhancement. (C) Time course of half-sarcomere length for each of the 50 half-sarcomeres.
(MOV)

Video S2 **Dynamic interactions between two dissimilar half-sarcomeres following stretch.** This is an animated version of Figure 8, depicting effects of stretch on two contracting, dissimilar half-sarcomeres connected in series. (A) The length-tension coordinates of the two half-sarcomeres are plotted during the simulation, with a tracer dot left every 0.1 seconds. The steady-state length-tension curves, which include passive and active tension, are shown for both half-sarcomeres, along with a dashed horizontal line indicating reference isometric tension. (B) Tension response to stretch (solid trace), with isometric reference (dashed line). (C) Time courses of average (black trace) and individual half-sarcomere lengths (red and blue traces). Colors correspond with those plotted in panel A.
(MOV)
